# Regulation of Heat Shock Proteins 27 and 70, p-Akt/p-eNOS and MAPKs by Naringin Dampens Myocardial Injury and Dysfunction *In Vivo* after Ischemia/Reperfusion

**DOI:** 10.1371/journal.pone.0082577

**Published:** 2013-12-06

**Authors:** Neha Rani, Saurabh Bharti, Mansi Manchanda, T. C. Nag, Ruma Ray, S. S. Chauhan, Santosh Kumari, Dharamvir Singh Arya

**Affiliations:** 1 Department of Pharmacology, All India Institute of Medical Sciences, New Delhi, India; 2 Department of Biochemistry, All India Institute of Medical Sciences, New Delhi, India; 3 Department of Anatomy, All India Institute of Medical Sciences, New Delhi, India; 4 Department of Pathology, All India Institute of Medical Sciences, New Delhi, India; 5 Department of Plant Physiology, Indian Agricultural Research Institute, Pusa, New Delhi, India; University of Otago, New Zealand

## Abstract

Naringin has antioxidant properties that could improve redox-sensitive myocardial ischemia reperfusion (IR) injury. This study was designed to investigate whether naringin restores the myocardial damage and dysfunction *in vivo* after IR and the mechanisms underlying its cardioprotective effects. Naringin (20–80 mg/kg/day, p.o.) or saline were administered to rats for 14 days and the myocardial IR injury was induced on 15^th^ day by occluding the left anterior descending coronary artery for 45 min and subsequent reperfusion for 60 min. Post-IR rats exhibited pronounced cardiac dysfunction as evidenced by significantly decreased mean arterial pressure, heart rate, +LVdP/*dt*_max_ (inotropic state), -LVdP/*dt*_max_ (lusitropic state) and increased left ventricular end diastolic pressure as compared to sham group, which was improved by naringin. Further, on histopathological and ultrastructural assessments myocardium and myocytes appeared more normal in structure and the infarct size was reduced significantly in naringin 40 and 80 mg/kg/day group. This amelioration of post-IR-associated cardiac injury by naringin was accompanied by increased nitric oxide (NO) bioavailability, decreased NO inactivation to nitrotyrosine, amplified protein expressions of Hsp27, Hsp70, β-catenin and increased p-eNOS/eNOS, p-Akt/Akt, and p-ERK/ERK ratio. In addition, IR-induced TNF-α/IKK-β/NF-κB upregulation and JNK phosphorylation were significantly attenuated by naringin. Moreover, western blotting and immunohistochemistry analysis of apoptotic signaling pathway further established naringin cardioprotective potential as it upregulated Bcl-2 expression and downregulated Bax and Caspase-3 expression with reduced TUNEL positivity. Naringin also normalized the cardiac injury markers (lactate dehydrogenase and creatine kinase-MB), endogenous antioxidant activities (superoxide dismutase, reduced glutathione and glutathione peroxidase) and lipid peroxidation levels. Thus, naringin restored IR injury by preserving myocardial structural integrity and regulating Hsp27, Hsp70, p-eNOS/p-Akt/p-ERK signaling and inflammatory response.

## Introduction

After an acute myocardial infarction (MI), early reperfusion therapy either by thrombolysis or primary percutaneous coronary intervention seems to be the most practical and widely practiced approach for myocardial salvage [1]. Nevertheless, reperfusion itself may paradoxically intensify further injury to ischemic myocardium, termed as “Ischemia-reperfusion (IR) injury” [1,2]. Several recent studies show that exaggerated production of reactive oxygen species (ROS) along with decreased endogenous antioxidant defense such as heat shock proteins (Hsp’s) are involved in the pathogenesis of myocardial IR injury [3-5]. Likewise, a large body of evidence demonstrates that over expression of Hsp27 and Hsp70 protects against IR-induced cardiac dysfunction and increases the resistance of the heart to ischemic injury [4,5]. Furthermore, in ischemia-reperfused hearts, ROS impaired cardiac functioning by decreasing NO bioavailability and/or by decreasing endothelial nitric oxide synthase (eNOS) expression/activity [6,7]. Of note, IkB kinase/nuclear factor-kappa B (IKK-β/NF-κB) and mitogen-activated protein kinases (MAPKs) including c-Jun N-terminal kinase (JNK) pathways are activated and extracellular signal-regulated kinase (ERK) suppressed in IR heart leading to apoptosis and necrosis of myocytes [6-9].

Naringin is a widely distributed bioflavonoid and polyphenolic compound predominantly found in grapefruits and related citrus herbs species. It possesses wide range of biological and pharmacological activities including antioxidant, anti-inflammatory, anti-apoptotic, anti-diabetic, cholesterol lowering, and hepatoprotective activity [10]. Owing to its ability to regulate oxidative stress, naringin has been shown to avert isoproterenol induced myocardial necrosis in rats [11]. In view of the above facts, naringin antioxidant potential has prompted us to scrutinize whether it is capable of exerting salutary effects in the left anterior descending (LAD) coronary artery ligation model of MI in rats.

Therefore, the present study has been designed for the first time to investigate (1) whether naringin, a powerful natural antioxidant, reverse cardiac dysfunction and morpho-histological changes in *in vivo* model of MI by alteration of the redox state through regulation of molecular chaperones Hsp27 and 70; or (2) whether this response is through regulation of p-Akt/p-eNOS signaling as well as reduced NO inactivation, and thereby increased NO bioavailability; or (3) whether this cardioprotective effects is through controlling the apoptotic, IKK-β/NF-κB and MAPKs signaling pathway.

## Materials and Methods

### Animals and Diet

Our work was conducted in accordance with the Indian National Science Academy (INSA) Guidelines for the use and care of experimental animals and approved by the Institutional Animal Ethics Committee of All India Institute of Medical Sciences (AIIMS), New Delhi, India (IAEC No. 593/11). Wistar albino male rats aged 6-8 weeks (160-180g, n=80) were kept in polypropylene cages, under controlled temperature (25±2°C), relative humidity (60±5%) conditions and subjected to natural photoperiod (light-dark cycle of 12:12 h). During the entire experimental period, the rats were allowed free excess to standard pellet diet (Ashirwad Industries Ltd.; India) and tap water *ad libitum*.

### Reagents

Naringin was procured from Sigma Chemical Company (St. Louis, MO, USA). It was dissolved in normal saline prior to its administration. All primary and secondary antibodies were procured from Cell Signaling Technology, USA except β-catenin and Bax which were purchased from Santa Cruz, Texas, USA and Bioss, MA, USA respectively. Rat Tumor necrosis factor-alpha (TNF-α) (Diaclone Tepnel Company, UK), Creatine Kinase isoenzyme-MB (CK-MB) (Spinreact, Spain), and lactate dehydrogenase (LDH) isoenzyme (Logotech, Delhi, India) kits were used.

### Experimental Protocol

Rats were divided into six groups viz. 

Group 1: Sham group (n=10)

Rats were administered normal saline orally (3mL/kg/day) for a period of 14 days. Subsequently, on 15^th^ day the experimental animals underwent the entire surgery, LAD coronary artery was exposed and suture was passed beneath it but was not subjected to ligation and reperfusion.

Group 2: IR control (n=15)

Rats were administered normal saline orally (3mL/kg/day) for a period of 14 days. Subsequently, on 15^th^ day experimental animals underwent 45 min LAD coronary artery ligation followed by 60 min reperfusion.

Groups 3-5: Naringin 20 + IR (n=15), Naringin 40 + IR (n=15) and Naringin 80 + IR (n=15)

Rats were administered naringin (20, 40and 80 mg/kg/day, p.o., respectively) for a period of 14 days. Subsequently, on 15^th^ day, experimental animals underwent 45 min LAD coronary artery ligation followed by 60 min reperfusion.

Group 6: Naringin *per se* (n=10)

Rats were administered naringin 80 mg/kg/day orally for a period of 14 days. Subsequently, on 15^th^ day, experimental animals underwent the entire surgery, LAD coronary artery was exposed and suture was passed beneath it but was not subjected to ligation and reperfusion.

### Induction of Myocardial IR Injury

Prior to surgical procedure, rats were anesthetized with pentobarbitone sodium (60 mg/kg, i.p.). Induction of myocardial IR injury was carried out by occluding LAD coronary artery for 45 min followed by 60 min reperfusion. After 15 min of stabilization period, hemodynamic and left ventricular functions such as mean arterial pressure (MAP), heart rate (HR), maximum speed of pressure development (±LVdP/*dt*_max_) and the left ventricular end-diastolic pressure (LVEDP) were recorded at 0, 5, 10, 15, 30, 45 min after coronary artery ligation and at 0, 5, 10, 15, 30, 45 and 60 min following reperfusion [6,7].

After completion of the reperfusion period, blood samples were withdrawn from the heart and serum was separated via centrifugation (Heraeus Biofuge, Germany) at 3000g for 5 min. Thereafter, animals were sacrificed under an overdose of anesthesia (pentobarbitone sodium 100 mg/kg, i.v.), and their hearts were excised and processed for histopathological, ultrastructural, biochemical and molecular studies.

### Infarct Size Determination

Monastral blue (0.5 mL/kg) was injected into the left atrium over 30 sec at the end of the reperfusion period to determine the *in vivo* area at risk. Thereafter rats were sacrificed; their heart was excised and kept at -20°C for 30 min for uniform sectioning of the tissue. Both unstained and stained blue dye masses of heart slices were incubated separately in 1% buffered (pH 8.5) triphenyltetrazolium chloride (TTC) for 20 minutes at 37°C for visualization and measurement of infarcted area [6].

### Biochemical Studies

A 10% heart homogenate was prepared in ice-chilled phosphate buffer (0.1M, pH 7.4) and an aliquot was used for the estimation of thiobarbituric acid substances (TBARS) [12] and reduced glutathione (GSH) [13]. In addition, the rest of the heart homogenate was then centrifuged at 3000g for 20 min at 4°C and the supernatant was used to measure Lactate Dehydrogenase (LDH), Glutathione Peroxidase (GSH-Px) [14], Superoxide Dismutase (SOD) [15] and Nitrite levels (NO) [16]. Moreover, Creatine Kinase-MB (CK-MB) activity and Tumor Necrosis Factor- alpha levels (TNF-α) were measured spectrophotometrically in serum.

### Western Blot Analysis

Heart tissues (40μg protein samples) were separated by sodium dodecyl sulphate-poly acrylamide gel electrophoresis, transferred to nitrocellulose membrane (MDI, Ambala, India) which was blocked for 2h with 5% bovine serum albumin or non fat dried milk and incubated for 12h at 4°C with primary antibody. The antibodies used were: SAPK/JNK, phospho-SAPK/JNK (Thr183/Tyr185), eNOS, phospho-eNOS (Ser1177), p44/42 MAPK (ERK ½) (137F5), phospho-p44/42 MAPK (ERK ½)(Thr202/Tyr204), Akt, phospho-Akt (Ser473), β-catenin, Hsp27, Hsp70, IKK-β (L570), NF-κB p65, Caspase-3, Bax, Nitrotyrosine, Bcl-2 and β-actin. The primary antibody was detected with HRP-conjugated anti-rabbit/anti-mouse secondary antibody. The antibody-antigen complexes were visualized using enhanced chemiluminescence kit (Thermo scientifc) under FluorChem M Protein imaging System (Bucher Biotec AG, Basel Switzerland) and were quantified by Bio-Rad Quantity One 4.4.0 software (BIO-RAD, Hercules, CA, USA). 

### Terminal Deoxynucleotidyl Transferase dUTP Nick End Labeling (TUNEL) Assay

TUNEL assays were performed using an *in situ* cell death detection kit, POD (Roche, Germany) following the manufacturer’s instructions.

### Histological and Ultrastructural Evaluation

Light and electron microscopic analysis of myocardial tissue were performed on the infarcted myocardium according to the method described in our previous papers [6,7]. The pathologist performing histopathological and ultrastructural analysis was masked to the treatment allocation.

### Immunohistochemistry (IHC) Analysis

IHC was performed using VECTOR ABC KIT, CA, USA. Briefly, slides were deparaffinized and tissue sections were hydrated through xylene and graded alcohol series. Slides were placed in pre-warmed citrate buffer (pH 6.0) for antigen retrieval, rinsed 3 times for 5 minutes in Tris Buffer Saline (TBS) and blocked in given kit serum solution for 45 minutes. Slides were then incubated overnight with primary antibody (Bax, Bcl-2 and Caspase-3; 1:500 dilution) at 4°C. Furthermore, slides were washed 3 times in TBS for 5 min and incubated in 3% H_2_O_2_ for 20 minutes to block the endogenous peroxidase activity. Slides were then washed 2 times with TBS and incubated with secondary antibody (1:200 dilution) for 45 minutes at room temperature. Slides were then again rinsed 3 times for 5 minutes with TBS and developed with 3,3'-diaminobenzidine. Slides were counterstained with haemotoxylin, mounted with DPX and visualized under microscope. 

### Statistical Analysis

The quantitative variables were expressed as mean ±S.D. Hemodynamic parameters were analyzed via repeated ANOVA test followed by *post hoc* Bonferroni test using Statistical Product and Service Solutions (SPSS) software package Version 11.5. One way ANOVA followed by *post hoc* Bonferroni test was used for evaluation of western blotting, biochemical parameters and histopathological grading. In both the cases, differences were considered statistically significant at P<0.05.

## Results

### Mortality

An overall mortality of 7.5% was observed during the study period. The animals died due to excessive bleeding or ligation of the coronary artery during surgery.

### Hemodynamic Parameters

**Figure 1:**
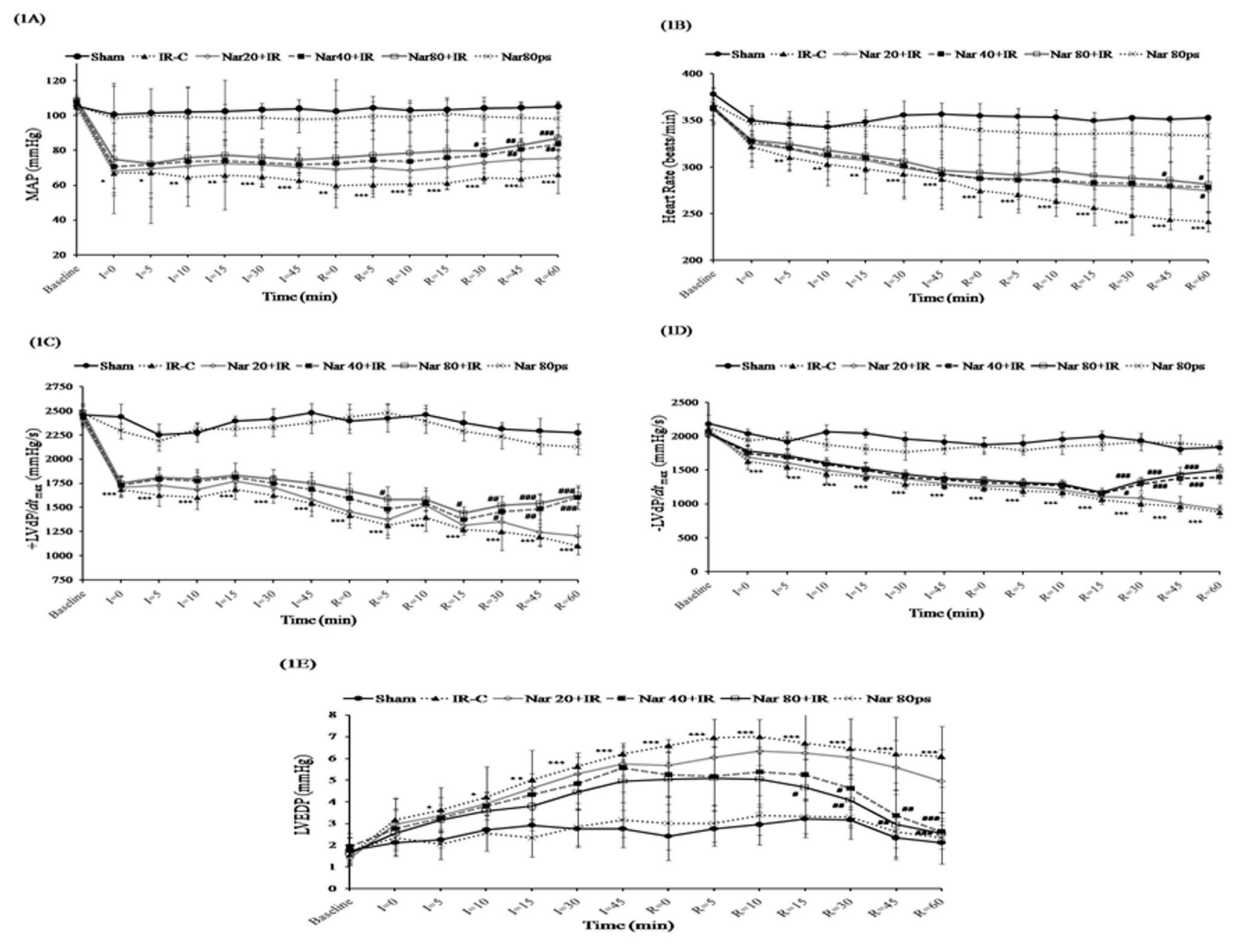
Effect of naringin on hemodynamic parameters following ischemia reperfusion. (A) Mean arterial pressure (MAP) (B) Heart rate (HR) (C) Maximal positive rate of left ventricular pressure (+LVdP/*dt*_max_) (D) Maximal negative rate of left ventricular pressure (-LVdP/*dt*_max_) (E) Left ventricular end diastolic pressure (LVEDP). Data are expressed as mean±S.D (n=6/group).^*^P<0.05,^**^P<0.01,^***^P<0.001 vs. sham and ^#^P<0.05, ^##^P<0.01, ^###^P<0.001 vs. IR-C.

Figure **1A-1E** exemplifies the effects of naringin on hemodynamic and left ventricular functions in experimental model of IR-induced myocardial infarction in rats. During IR challenge, we observed significantly (P<0.001) decreased MAP, HR, ±LVdP/*dt*_max_ and increased LVEDP at almost all the time intervals as compared to sham group. In comparison to IR control group, pretreatment with naringin (40 and 80mg/kg/day) for 14 days prevented fall in MAP values at every time point of IR period but a significant (P<0.01) increment was observed at 45, 60 min of the reperfusion period (Figure **1A**). Naringin treatment did not significantly restore HR throughout ischemic duration as compared to IR control group. However, restoration in HR value reached statistical significance (P<0.05) at 40 (60 min of reperfusion) and 80 mg/kg/day dose (45 and 60 min of reperfusion) (Figure **1B**). Concurrently, naringin 40 and 80 mg/kg/day also significantly (P<0.01) restored ±LVdP/*dt*_max_ and decreased LVEDP value during late phase of reperfusion period (Figure **1C-1E**).

### Protein Expressions

**Figure 2:**
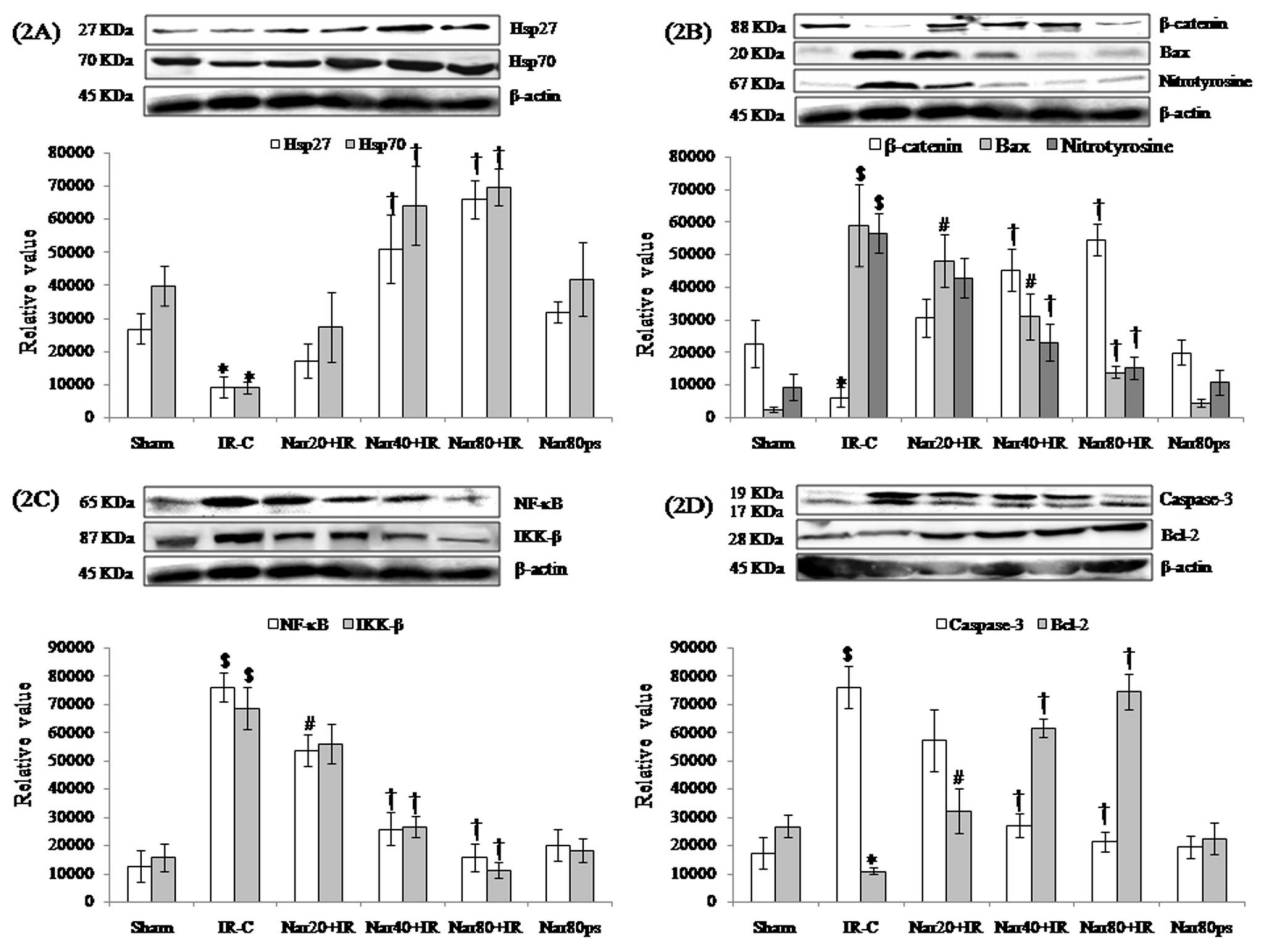
Effect of naringin on protein expressions in different experimental groups. (A) Hsp27 and Hsp70 (B) β-catenin, Bax and Nitrotyrosine (C) NF-κB and IKK-β (D) Caspase-3 and Bcl-2. All values for protein expressions are expressed as mean±S.D (n=3/group). ^*^P<0.05, ^$^P<0.001 vs. sham and ^#^P<0.01, †P<0.001 vs. IR-C.

Western blot analysis showed that naringin (40 and 80 mg/kg/day) significantly (P<0.001) increased β-catenin, Hsp27 and 70 expressions as compared to IR control group (Figure **2A and 2B**). Moreover, naringin (40 and 80 mg/kg/day) mediated inhibition of IR injury is also linked with suppression of inflammatory signaling as we observed significantly (P<0.001) decreased IKK-β and NF-κB expression (Figure **2C**). 

To dissect the role of apoptotic signaling pathway in ischemia reperfusion model of myocardial infarction we next evaluated the protein expression of apoptotic and anti-apoptotic proteins through both western blotting and immunohistochemistry. As anticipated naringin significantly (P<0.001) decreased Bax expression, caspase-3 expression, TUNEL positivity and increased Bcl-2 expression in IR treated rats (Figure **2B, 2D**, 4F, 4K and 5A3-5F6). 

**Figure 3:**
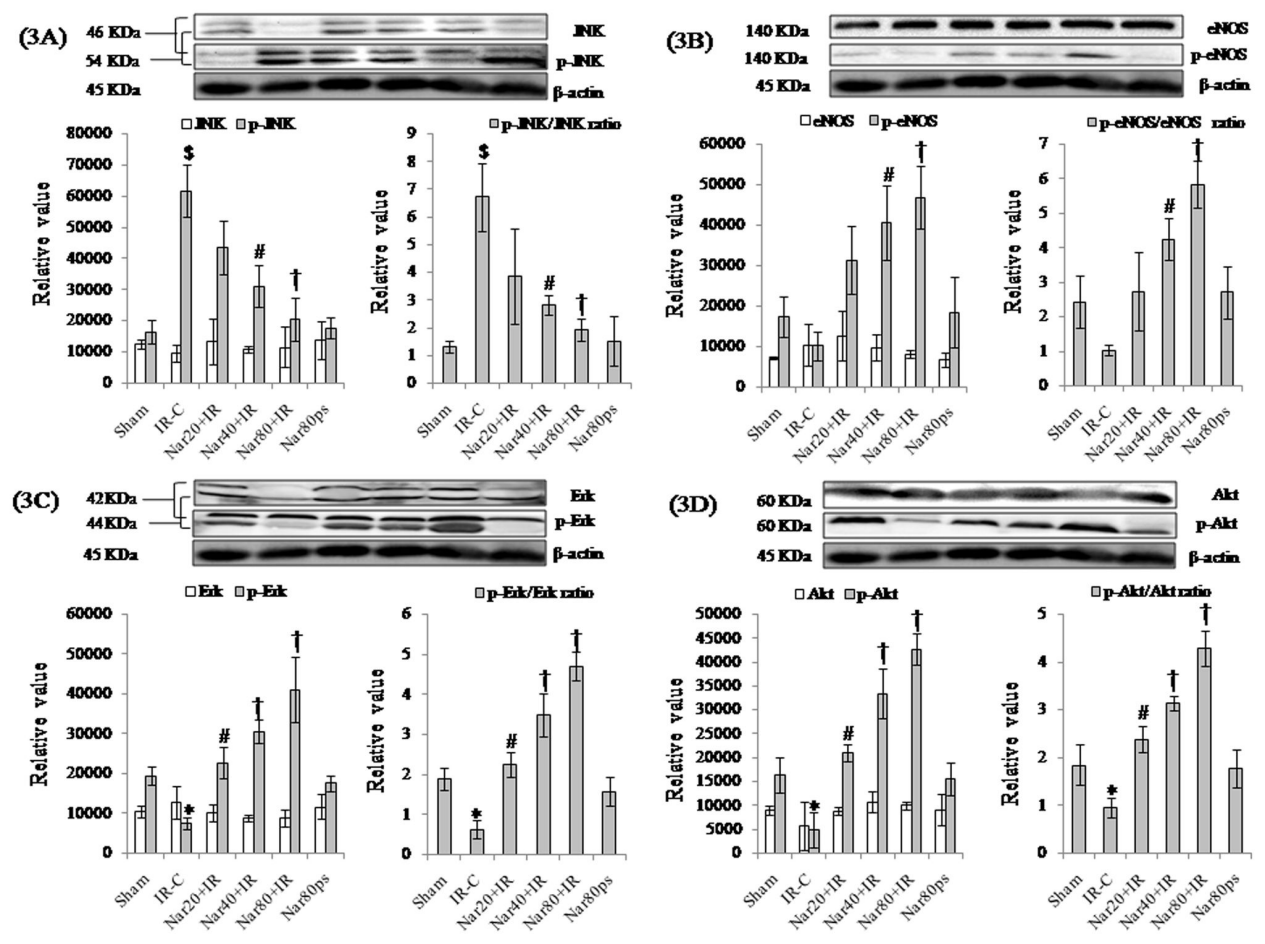
Effect of naringin on protein expressions in different experimental groups. (A) JNK, p-JNK and p-JNK/JNK ratio (B) eNOS, p-eNOS and p-eNOS/eNOS ratio (C) ERK, p-ERK and p-ERK/ERK ratio (D) Akt, p-Akt and p-Akt/Akt ratio. All values are expressed as mean±S.D (n=3/group). ^*^P<0.05, ^$^P<0.001 vs. sham and ^#^P<0.01, †P<0.001 vs. IR-C.

Furthermore, to determine whether myocardial salvaging effect of naringin is governed by the recruitment of Akt/eNOS signaling, we next assessed the phosphorylation of these proteins in the failing (IR) as well as recovering (naringin) myocardium. Surprisingly, we did not observe any significant change in Akt and eNOS expressions in different experimental groups, though we observe significantly (P<0.01) elevated expressions of p-Akt, p-Akt/Akt ratio, p-eNOS and p-eNOS/eNOS ratio as well as decreased NO inactivation to NT, thereby, supporting the role of NO pathway in naringin mediated cardioprotection. Interestingly, the expression of MAPKs including JNK and ERK were not significantly different in either of the treatment groups. However, naringin significantly (P<0.01) normalized the phosphorylation of JNK and p-JNK to JNK ratio in IR treated rats. Additionally, naringin also amplified the phosphorylation of ERK and the ratio of p-ERK to ERK in IR treated rats (Figure **2B** and **3A**-**3D**). 

### Biochemical Parameters

**Figure 4:**
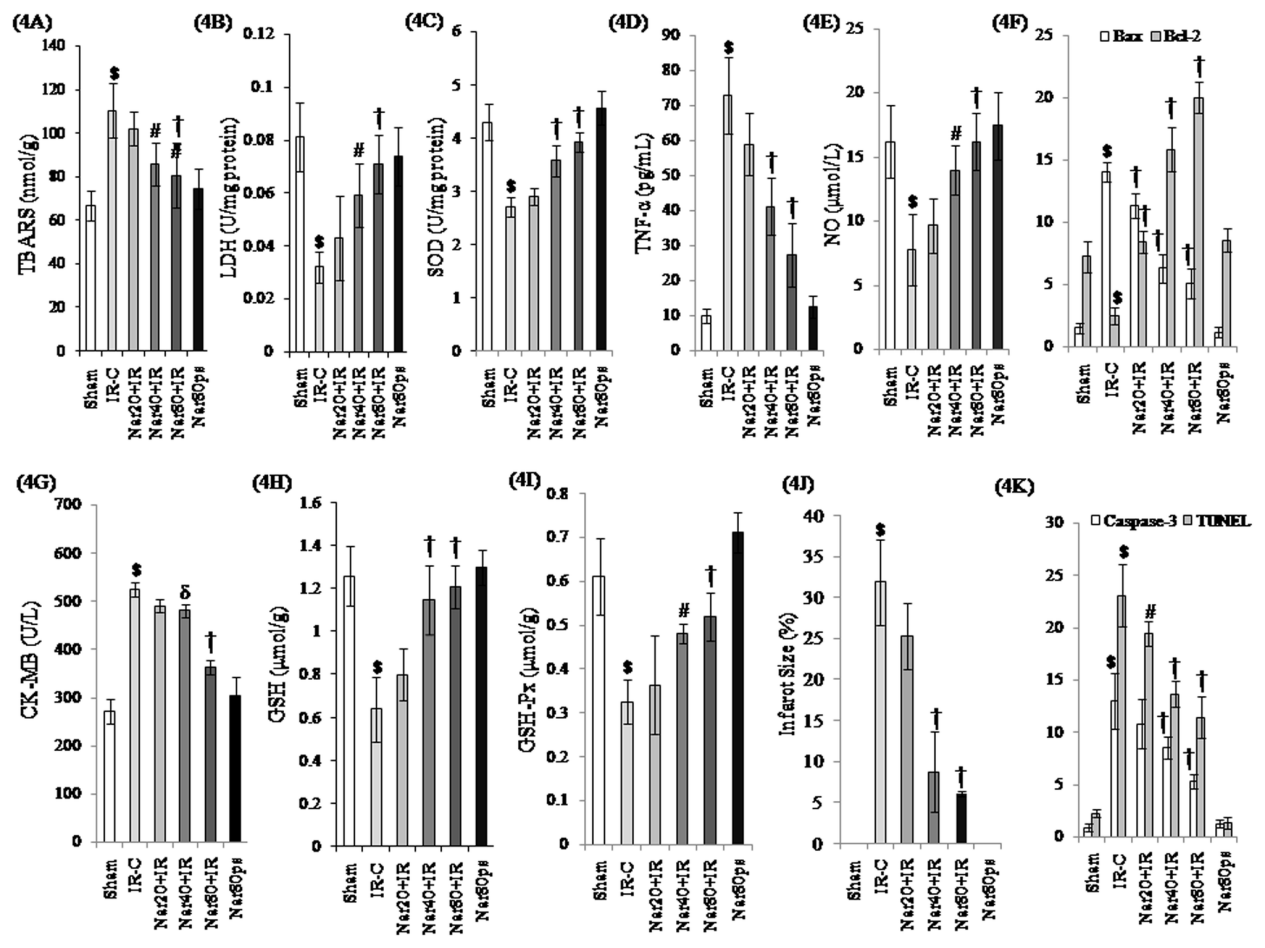
Effect of naringin on biochemical findings in different experimental groups. (A) TBARS: Thiobarbituric acid reactive substances; (B) LDH: Lactate dehydrogenase; (C) SOD: superoxide dismutase; (D) TNF-α: Tumor necrosis factor-alpha; (E) NO: Nitric oxide; (F) immunohistochemistry quantification of Bax and Bcl-2; (G) CK-MB: Creatine kinase-MB isoenzyme; (H) GSH: Reduced glutathione; (I) GSH-Px: Glutathione peroxidase; (J) Infarct size and (K) immunohistochemistry quantification of Caspase-3 and cardiomyocyte TUNEL positive nuclei. Data are expressed as mean±S.D (n=6/group for all except for infarct size which is n=3/group).^*^P<0.05, ^$^P<0.001 vs. sham and ^δ^P<0.05, ^#^P<0.01, †P<0.001 vs. IR-C.

Figure 4A 4E and 4G-4I illustrates the activities of lipid peroxidation, cardiac injury marker enzymes, antioxidant activities, NO and TNF-α levels in experimental model of IR-induced myocardial injury in rats. IR control rats showed significantly (P<0.001) reduced LDH, SOD, GSH, GSH-Px and NO content along with increased TBARS level in the myocardium as compared to sham group. Furthermore, significant (P<0.001) rise in serum CK-MB isoenzyme activity and TNF-α levels were observed in IR control group as compared to sham group. Interestingly, naringin (40 and 80 mg/kg/day) significantly restored NO, TNF-α, GSH and TBARS levels and LDH, CK-MB, SOD and GSH-Px enzymatic activities in comparison to the respective IR control group. 

### Histopathological and Ultrastructural Assessment

**Figure 5:**
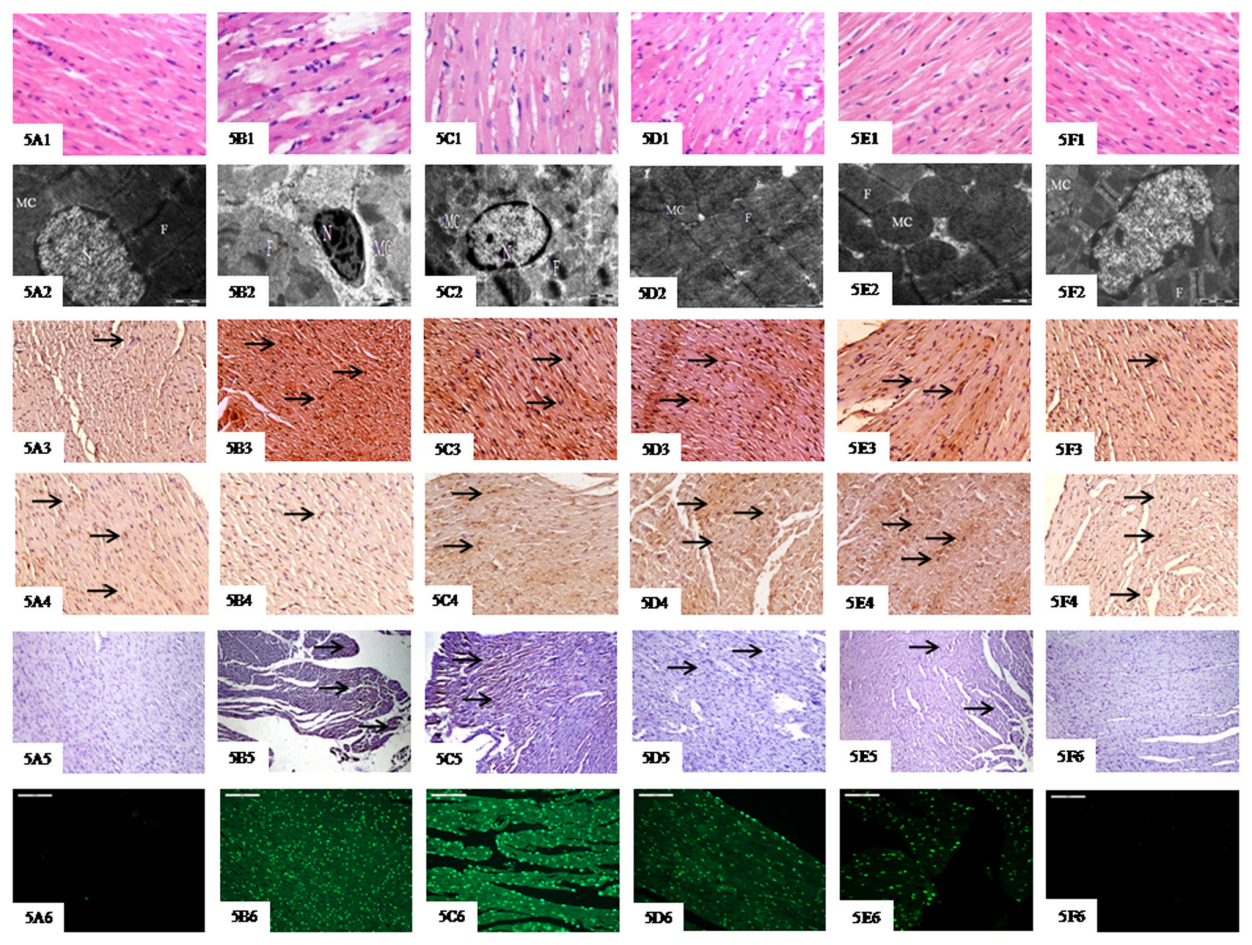
Effect of naringin on light microscopic changes (A1-F1, 20X, Scale bar 50μm), electron microscopic changes (A2-F2, 4000X, Scale bar 1μm), Bax immunohistochemistry (A3-F3, 10X, Scale bar 50μm), Bcl-2 immunohistochemistry (A4-F4, 10X, Scale bar 50μm), Caspase-3 immunohistochemistry (A5-F5, 10X, Scale bar 50μm) and TUNEL positivity (A6-F6, 20X, Scale bar 100μm) in different experimental groups. Sham group (5A1-5A6); IR-C (5B1-5B6); IR + naringin 20, 40 & 80 mg/kg/day respectively (5C1-5C6, 5D1-5D6 and 5E1-5E6); and naringin 80 mg/kg/day *per* *se* (5F1-5F6). Data are expressed as mean±S.D (n=6/group). **^*$*^**P<0.001 vs. sham and ^#^P<0.01, †P<0.001 vs. IR-C. N: nucleus; MC: mitochondria; F: myofibrils.

Figure **5A1** illustrates light micrograph features of sham group showing normal architecture of myocardium. In contrast, IR group showed cardiomyocyte membrane damage with extensive myonecrosis, marked edema and inflammatory cell infiltration (Figure **5B1** and Table **1**). Fourteen days pretreatment with naringin (40 and 80 mg/kg/day) resulted in significant structural improvement as evidenced by decreased myonecrosis, edema and inflammatory cell infiltration in myocardium, the effect being most pronounced at 80 mg/kg/day (Figure **5C1-5E1** and Table **1**). 

**Table 1:** Effect of naringin on grading of histopathological changes in different experimental groups (n=6/group).

**Groups**	**Myonecrosis**	**Edema**	**Inflammation**
Sham	-	-	-
IR-C	+++	++++	+++
Nar20+IR	++	+++	++
Nar40+IR	++	++	+
Nar80+IR	+	+	+
Nar80ps	-	-	-

Score (-): Absence of any myonecrosis, edema and inflammation; Score (+): Focal areas of myonecrosis, edema and inflammation; Score (++): Patchy areas of myonecrosis, edema and inflammation; Score (+++): Confluent areas of myonecrosis, edema and inflammation; Score (++++): Massive areas of myonecrosis, edema and inflammation (n=6/group).

Figure **5A2** illustrates ultrastructural sections of sham group showing normal myofibrils, mitochondrial structure with active nucleus and uniformly scattered glycogen granules. Subsequent to IR challenge, significant myofibrillar derangement and degeneration; swollen and irregular mitochondria with disrupted cristae and chromatin condensation were observed (Figure **5B2**). Electron photomicrographs of naringin (20 mg/kg/day) treated group revealed structural damage comparable to IR control group (Figure **5C2**). Fourteen days pretreatment with naringin (40 and 80 mg/kg/day) revealed normal myofibrillar ultrastructure and mild separation of the mitochondrial cristae without swelling and vacuolation, though, improvement was most prominent at its highest dose (Figure **5D2 and 5E2**). However, naringin per se (80 mg/kg/day) in light and electron micrographs showed same myocardial integrity as that of sham group (Figure **5F1 and 5F2**). 

### Irreversible Ischemia Reperfusion Injury Assessment

Figure **4J** shows percent infarcted size after TTC staining. Ischemia of 45 min and reperfusion of 60 min was accompanied with development and progression of irreversible myocardial infarction. Pretreatment with naringin 40 and 80 mg/kg/day significantly diminished the infarct size (8.749±4.889 and 6.015±0.32 vs. 31.84±5.19, P<0.001, respectively) in comparison to IR group.

## Discussion

In this paper we focused on the cardioprotective potential of naringin and its underlying molecular mechanism/s. Our results identify naringin as a cardioprotective agent as it reduced myocardial infarct size; preserved myocyte structural integrity and decreased LVEDP and tended to improve ±LVdP/*dt*_max_ *in vivo* after IR. This myocardial salvaging effect of naringin is primarily mediated through attenuation of oxidative stress by improving endogenous antioxidant defense system via amplified expression of β-catenin, Hsp27 and Hsp70 and interruption of inflammatory signaling through TNF-α/IKK-β/NF-κB suppression. Interestingly, naringin increased Akt and eNOS phosphorylation and augmented myocardial NO production. Importantly, regulation of MAPKs including inhibition of JNK, as well as activation of ERK by naringin was also involved in attenuation of IR injury. Further, naringin anti-oxidant and anti-inflammatory finding firmly corroborated with its anti-apoptotic potential as it precludes myocyte apoptotic cell death through increasing Bcl-2 expression and decreasing TUNEL positivity, Bax and caspase-3 expression. Thus, our findings identify a novel mechanism whereby naringin could be an effective candidate against IR induced myocardial damage and dysfunction.

In our study, myocardial IR injury was produced by occluding LAD coronary artery for 45 min and subsequent 60 min reperfusion. Our findings as well as previous studies have demonstrated that global ischemia followed by reperfusion in rat hearts is associated with depressed contractile function and increased preload as reflected by decreased MAP, HR, (±) LVdP/*dt*_max_ and increased LVEDP [6]. Fourteen days pretreatment with naringin (40 and 80 mg/kg/day) resulted in preservation of myocardial function as evidenced by significant improvement in inotropic (+LVdp/*dt*_max_, myocardial contraction) and lusitropic (–LVdp/*dt*_max_, myocardial relaxation) states, LVEDP (preload), MAP and HR. The plausible reason for the preventive effect of naringin could be an increased expression of Hsp27/Hsp70 or phosphorylation of Akt/eNOS/ERK, as their increased expression stabilizes troponins I and T, and activates SERCA2a channel, leading to diminished cytosolic Ca^2+^ overload, improved contractile function and increased resistance to ischemic injury [4,5,17-19]. Additionally, the other mechanisms could be due to direct inhibition of Ca^2+^ influx, or activation of K^+^ influx [20,21]. A recent study published by Alam and co workers further supports our hypothesis as they have shown that naringin improves diet-induced cardiovascular dysfunction and obesity in high carbohydrate, high fat diet-fed rats [22]. 

Myocardial IR injury may intensify pathological processes that contribute to ROS generation, disturbances in cation homeostasis, and depletion of cellular energy stores, which may eventually result in myocardial necrosis and dysfunction. Substantial evidences suggest that ROS-induced oxidative stress is considered a major causative factor in the pathogenesis of myocardial IR injury [1,2,6,23]. As demonstrated here, IR resulted in significant increase in TBARS level along with decreased endogenous antioxidants such as GSH, SOD and GSH-Px activities, validating the presence of increased oxidative stress in IR control group. Additionally, naringin significantly augmented myocardial endogenous antioxidant defense i.e. GSH content, SOD and GSH-Px activities and reduced lipid peroxidation, thereby protecting cardiomyocytes from death. These data allow us to hypothesize that enhanced myocardial SOD, GSH and GSH-Px activities by naringin could be an important mechanism of its cardioprotection.

Substantial literature suggests that molecular chaperones such as the Hsp27 and 70 actively participate in an array of cellular processes by mediating protein folding and transport, preventing protein aggregation, denaturation and degradation as well as promoting damaged protein elimination [24]. In addition, in IR hearts, Hsp27 and 70 maintain oxidative metabolism, protect mitochondria from ROS, repair ion channels and critical structural cytoskeletal proteins, suppress pro-inflammatory cytokines, NADPH oxidase and apoptosis, and have an inverse correlation with infarct size and myocardial dysfunction [3-5,24-26]. In agreement with this, our results suggest that one of mechanism by which naringin attenuates IR injury could be due to overexpression of Hsp27 and 70, as we observed significantly increased expressions of these accompanying amelioration of IR injury in naringin treated rats.

It has been reported that the progression of IR injury is characterized by an inflammatory response in which increased TNF-α production and/or IKK-β/NF-κB activation along with neutrophil and monocyte/macrophage infiltration play an important role. In IR hearts, activated neutrophils release ROS and proteases, and activated monocytes/macrophages discharge inflammatory cytokines which further trigger TNF-α/IKK-β/NF-κB signaling. Furthermore, multiple studies have shown that increased TNF-α production and/or activation of IKK-β/NF-κB are the key mediators of inﬂammation, oxidative stress and vascular endothelial dysfunction in ischemia reperfusion model of myocardial infarction [1,2,27,28]. Notably, we observed significant reduction in serum TNF-α level, IKK-β/NF-κB expression and inﬂammatory cell recruitment into the ischemic myocardium in naringin treated rats, suggesting a potential mechanism by which naringin protects the heart from IR injury. 

Several lines of evidence suggest that NO is a fundamental determinant of cardiovascular homeostasis. It regulates systemic blood pressure, left ventricular function, vascular remodeling, angiogenesis, reduces calcium overload, ROS production, apoptosis and neutrophil-associated injury in IR heart [29]. Production of NO is regulated by serine/threonine kinase p-Akt-dependent phosphorylation of eNOS [6,7]. Interestingly, our findings demonstrate that naringin significantly augmented p-Akt/p-eNOS expression and myocardial NO. Meanwhile, we also observed concomitant decrease in NT in heart homogenate, a surrogate marker for ROS mediated NO inactivation. Therefore, it is possible to explain that the infarct-size limiting effect of naringin in our experiment is partly due to increased NO production by p-Akt-dependent activation of eNOS. Moreover, increased myocardial NO bioavailability is also linked with its antioxidant activity as it attenuated NO inactivation to NT.

MAPKs are multifunctional regulators that play a key role in a number of biological processes in heart including cell proliferation, survival, apoptosis, actin reorganization and cytokine production [30]. Cardioprotection rendered by JNK inhibition and ERK activation has been supported by a number of studies [30-32]. The increased phosphorylation of JNK contributes to the activation of the intrinsic apoptotic pathway in response to cellular stress. In contrast, increased ERK phosphorylation showed anti-apoptotic activity. One intriguing observation in the present study is that compared to IR control, naringin treated rats showed significantly decreased JNK phosphorylation (inhibition) and a concomitant increase in ERK phosphorylation (activation). These data provide strong support for the premise that naringin attenuates IR injury, in part via modulation of JNK and ERK phosphorylation in cardiomyocytes.

Previous studies have shown that IR results in substantial induction of apoptosis, and susceptibility to cardiac dysfunction depends on the degree of myocyte apoptosis [6,7,33]. Importantly, in our present study naringin showed anti-apoptotic activity as it up-regulated Bcl-2 expression and attenuated TUNEL positivity and Bax and caspase-3 expression in IR heart. Therefore, it is conceivable that attenuation of myocyte apoptosis by naringin might be due to direct regulation of Bcl-2 and Bax/caspase-3 and/or indirectly via modulation of p-Akt/p-eNOS/NO signaling, MAPKs and endogenous antioxidant activity. Our data is in corroboration with a recent finding where naringin abolishes high glucose-induced cardiomyocyte apoptosis by attenuating mitochondrial dysfunction and modulating the activation of the p38 signaling pathway [34]. 

Subsequent to IR challenge, CK-MB and LDH released from damaged myocytes enter the circulation. Thus, leakage of these enzymes provides substantial evidence of sarcolemmal integrity disruption [1,2,6,7]. Notably, naringin significantly increased myocardial LDH level and decreased serum CK-MB activity. These findings suggest that naringin protects the integrity of sarcolemma, which restricts the leakage of these marker enzymes from myocardium. Further, TTC staining of naringin treated rats showed significant reduction in necrotic area. Similarly, morphological analysis of heart showed that naringin significantly preserved myocardial structural integrity as evidenced by absence of myonecrosis, inflammation, edema and normal myofibrillar ultrastructure in IR heart.

In conclusion, our present work demonstrates that naringin may serve as a promising cardioprotective agent, which could effectively protect the myocardium against IR-induced myocardial damage and could be used as add on therapy in patients for the secondary prevention of post myocardial infarction or for the primary prevention in patients who are having multiple risk factors like family history of early coronary artery disease, hypertension, hypercholesterolemia and diabetes [10,22,34]. Our results indicate that augmentation of endogenous antioxidant activity, upregulation of p-Akt/p-eNOS/NO signaling and suppression of IKK-β/NF-κB along with modulation of MAPKs by naringin synergistically contribute to the alleviation of irreversible IR injury and promote cardiomyocyte survival. To examine the hypothesis that naringin may be safe and effective approach to preventing and treating MI, further experimental and clinical studies are warranted.
